# Dental Hygienist and Preventive Dentistry

**Published:** 1916-09

**Authors:** 


### THE DENTAL HYGIENIST AND PREVENTIVE DENTISTRY.

The movement to train young ladies to clean and polish the surfaces of teeth, and legalize their performance of this work, has met with some degree of favor in certain localities. This we believe to be a mistake and quite inimical to the best interests of the public and dental profession. In its support the sponsors for the movement claim the urgent need for oral cleanliness and a public requirement for services which can not be supplied by the dental profession at the present time.

We must bear in mind that oral health means something more than the periodic visitation to a dental office for prophylactic treatment. Possibly the greater element entering into the prevention of dental diseases will be found to be the daily conduct of the individual concerning those habits of living that make for general health and the proper *daily* care of the mouth. Any movement that in any way tends to shift the responsibility for cleanliness from the individual to some other person—whether it be dentist or so-called hygienist—is decidedly a retrograde step.

People must be educated to take the necessary time to keep their mouths clean and healthy. The ordinary individual realizes that he must keep his body in a clean and hygienic condition and quite willingly assumes the burden. And why not the mouth? Under modern conditions of living oral cleanliness has assumed a most important position. The technique of cleansing requires much more time than the average individual now gives to that task. A great many, through ignorance of the subject or through a lack of appreciation of its importance, neglect the hygiene of the mouth completely. The solution is public education and instruction—particularly in the schools. What the dentist needs more than the co-operation of the suggested professional hygienist is the co-operation of the individual patient. The dental hygienist idea might, however, appeal to the "idle rich"—if there be such—who may be too *idle* to clean their teeth and *rich* enough to pay the hygienist to perform that personal service.

We venture the opinion that if the individual give proper exercise and proper care to his teeth, he will require very little special prophylactic treatment, and, in the majority of cases, develop an immunity to dental disease.

If these young women are to give prophylactic treatment, including oral hygiene instruction, the examination of the mouth will be naturally included, and this in turn will naturally lead to the giving of advice. The patient will thus be deprived of the services of the dentist in some of the most important phases of dental practice. It seems almost incredible that there are those in the profession who, having regard to the best interests of their patients, would be satisfied to allow such conditions to obtain.

The oral hygienists of Connecticut recently held a convention, the programme of which, it was reported, "would do credit to any dental society, the principal address being delivered by Dr. F. C. Noyes upon the subject of the peridental membrane." Some rapid progress (?). But how can prophylaxis be intelligently performed without a knowledge of the histology of the tooth and the surrounding parts. How can prophylaxis be intelligently performed without an intimate knowledge of dental anatomy and the many pathological conditions found in the mouth?

A young lady trained as an efficient assistant to the dentist in the operating room renders most valuable aid to the dental surgeon and increases output of service materially. In place of schools for dental hygienists, we should have schools for dental assistants, so that there might be available to every dentist a trained assistant who would be "an extra pair of hands," and enable the dentist to better meet the demands that are made upon him. If dentists stopped puttering in the office over the hundreds of little things that could be better done by a trained assistant, there would be ample time for the more important services, namely, prophylaxis and instruction.—*Oral Health.*

